# Challenges in disclosure of adverse events and errors in surgery; perspectives from sub-Saharan Africa

**Published:** 2012-07-24

**Authors:** Abdulrasheed Ibrahim, Ekundayo Stephen Garba, Malachy Eneye Asuku

**Affiliations:** 1 Department of surgery, Ahmadu Bello University Teaching Hospital P.M.B 06 Shika Zaria, Nigeria

**Keywords:** Challenges, errors, adverse events, surgery, sub-Saharan Africa

## Abstract

Surgery in sub-Saharan Africa is widely known to be done against a background of poverty and illiteracy, late presentation with complicated pathologies, and a desperate lack of infrastructure. In addition, patient autonomy and self determination are highly flavored by cultural practices and religious beliefs. Any of these factors can influence the pattern and disclosure of adverse events and errors. The impact of these in the relationships between surgeons and patients, and between health institutions and patients must be considered as it may affect disclosure and response to errors. This article identifies the peculiar socioeconomic and cultural challenges that may hinder disclosure and proposes strategies for instituting disclosure of errors and adverse events services in Sub-Saharan Africa.

## Introduction

Surgery occurs in every setting in sub-Saharan Africa and the burden of unmet surgical need has increased greatly the demand for surgical intervention [1]. It is a given that all health care professionals involved in the care of surgical patients have the best of intentions and endeavor to ensure patient safety while providing quality health care [2]. It is however also well recognized that surgery although undoubtedly beneficial, can have potentially harmful effects on patients [3]. Unintended injury or complication or an undesirable outcome from the expected course of the disease leading to a prolonged admission, disability at discharge or death is defined as an adverse patient event in surgery. An error is defined as an adverse event or near miss that is preventable with the current state of medical knowledge [3–6].

Disclosure of adverse events and errors is desired by patients, endorsed by ethicists and professional organizations, and increasingly required by regulatory and government bodies [7]. Indeed, an important indicator of patient safety within a hospital is the documented rate of occurrence of adverse events and its disclosure during the course of inpatient admission [3, 4, 6]. Studies conducted in various developed countries have reported adverse patient events to occur in 3–30% of hospital admissions with permanent disability or death rates of about 0.4-0.8%. In developing countries, a death rate of 5-10% for major surgery is reported. Nearly half of the adverse events in these studies were identified as preventable [1].

As initiatives to improve outcome following surgical intervention move forward in low resource settings, there is a growing attention to disclosure of adverse events and errors in surgery. This is relatively recent in many parts of Sub-Saharan Africa and there is a paucity of both data and perspective [8, 9]. Thus, it is important to identify the peculiar socioeconomic and cultural challenges that may hinder disclosure and propose the best strategies for instituting disclosure of errors and adverse services in health care institutions in Sub-Saharan Africa.

## Patient challenges

Surgery in sub-Saharan Africa is widely known to be done against a background of the twin problems of poverty and illiteracy. Most patients live in the rural and semi urban areas and are not able to get to a hospital quickly in an emergency. The enormous cost of emergency care is likely to discourage the poor from accessing life-saving surgery [10].These patients are malnourished, cannot afford the cheapest medicines and are also reluctant to travel long distances for routine checkups and screenings in centralized services of tertiary health institutions in the urban area [10, 11]. The effects of these is that patients present when complications may have developed and so surgeons have to deal with advanced and complicated surgical pathology in routine clinical practice. These socio-economic patient issues also means that some patients are unlikely to afford multiple interventions that may be necessary as part of their care [11, 12] In many countries in sub-Saharan Africa, health insurance schemes are in a tragic state; Depending on the design, people are either unable to pay for the schemes, or the schemes are unable to pay for the envisaged services [13]. Endemic cultural practices poses significant challenges to patient care as patients attribute their health problems to religious beliefs, supernatural causes or try alternative actions that may be more harmful [12].

### Proposed action

Just as public-health interventions and educational projects have greatly improved maternal and neonatal survival in sub-Saharan Africa, so might analogous efforts in disclosure of adverse events and errors improve surgical outcome and quality of care [1]. All patients who are identified as high risk factors for a bad outcome following surgery, should have processes of care that will increase the probabilities of a good outcome [14]. These factors will affect the patients’ pathway from the initial pre-operative clinic to the final post-operative discharge clinic and should be communicated clearly to the patient and his family [15]. Patients need to know the specific implications for their subsequent care so they can make informed decisions based on the assessment of the surgeon. When adverse events or errors occur, patients should have a sincere apology from the surgeon or other health care professionals involved. A sincere apology is a powerful communication that recognizes the suffering of a patient and indicates the remorse of the professional in causing harm. Patients and their families should be informed of how the health care professional and the institution will learn from the error and prevent a similar event from happening to another patient in the future. Often patients feel that if something good can come from their misfortune, it will help them feel better about a bad situation [8].

## Surgeon challenges

Most surgeons in Sub-Saharan Africa are subjected to various stressful factors which can influence their performance in decision making, team interaction and technical skill. Work related stresses include fatigue due to sleep deprivation, a high workload, long commutes and time constraints [15]. Personal stresses may also include fatigue as a result of poor personal fitness or health, partner relationship problems, family illnesses and difficulties and financial strains. All these stressful factors influence the cerebral cortex in performing technical tasks and may increase the risk of adverse events and errors by surgeons [15, 16]. In addition, efforts to encourage disclosure of adverse events and errors to patients face multiple barriers, including discomfort in facing angry patients and their families, concern about potential damage to their reputation and more recently the fear of malpractice litigation. Furthermore, most surgeons have not received a formal training in disclosure. Overcoming this training deficit requires understanding how surgeons currently disclose errors and no studies have investigated how surgeons disclose errors in sub-Saharan Africa [7].

### Proposed action

Surgeons-in-training learn how to cope with adverse events and errors through denying it by viewing the practice of surgery as an art with gray areas, discounting it by blaming the hospital, superiors or subordinates, or the patient; and when they can no longer deny or discount error, distancing it-concluding that all surgeons’ make mistakes anyway, but usually do the best they can [4]. Undergraduate and post graduate surgery program coordinators should incorporate disclosure of adverse events and errors into the patient safety curriculum. Surgeons need to be taught to use this knowledge as a tool for reducing error and not an excuse for accepting it. The communication skills of surgeons should emphasize ways to disclose errors to their patients. In addition, more explicit attention should be paid to the impact of cultural differences in the relationships between surgeons and patients, and between medical institutions and patient cultures, which may affect the course of treatment and the response to adverse events and errors [4].

## Hospital administration challenges

Most government hospitals in Sub-Saharan Africa are overcrowded with long outpatient queues. There are also queues for investigations because the X-ray machine, the scanner, or autoanalyser is not functioning. The waiting time may range from 1 month for obstructive jaundice due to cancer, to 6 months for hernia repair or reconstruction for post burn flexion contracture of the digits of the hand. In the surgical ward, with probably only one nurse in a ward of about 25 or more patients, much of the nursing care is provided by family members and relatives, who also have to go to buy not only the patient′s food but also drugs, sutures, intravenous fluids, or anything else that is in short supply [10, 16]. The operating room would be unrecognizable to many from developed nations and still has the capacity to shock and dismay those who have been somewhat prepared for it. Broken or unavailable equipment, out-of-date or unavailable sutures and medications, and a desperate lack of essential items such as gloves, dressings, and scrub solutions are ubiquitous. Surgical teams have to manage with intermittent supplies of electricity, and many facilities do not have the funds to buy generators to manage the outages. Water supplies can also be erratic [10].

### Proposed action

**Figure 1:**
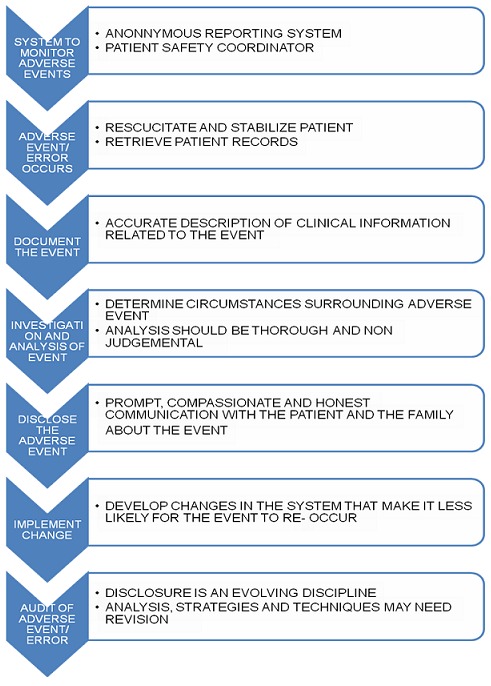
An algorithm for managing adverse events and errors

**Figure 2:**
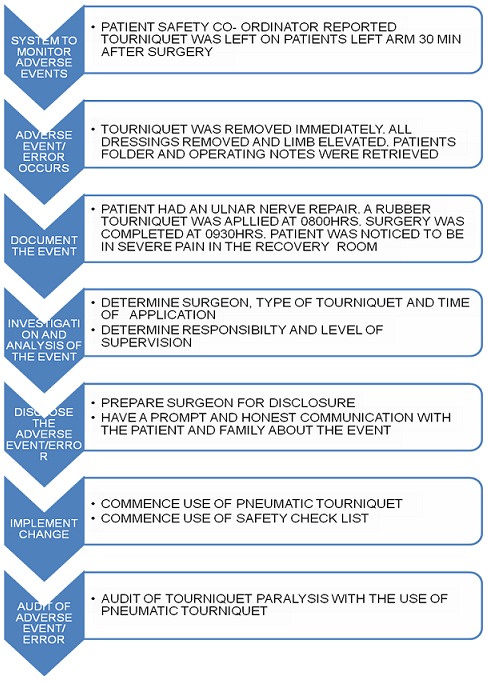
An algorithm illustrating an example for managing an adverse event (tourniquet paralysis) following prolonged application of a tourniquet

Against the background of severe shortages, any of these factors can influence the pattern of preventable adverse events and errors. A local epidemiological approach is suggested, in which institutions assess their local environment, identify specific problems, and allocate corrective resources to local priorities. There is thus a good chance that the problems and the solutions will match, resulting in the allocation of scarce resources to important problems and away from minor problems [4, 17]. Hospital administrators will also need to develop training programs and workshops on disclosure of adverse events and errors and provide support and counseling to surgeons to help them cope with their mistakes [4]. All Hospitals and surgical departments should establish an error disclosure unit saddled with the responsibility of full disclosure. The unit will develop a written institutional protocol including an explicit statement that there was an error, details of what went wrong and why the adverse event or error occurred [8]. Anonymous reporting system should be established to collect and analyze information about adverse events and errors, emphasizing relevant information about both the processes of care and the consequences of those actions. Figure 1 and Figure 2 [14, 18]. A recent advance in the field of improving surgical patient safety is the World Health Organization's (WHO) surgical safety checklist which consists of 19 basic items directly pertaining to pre- and post-operative patient care. A prospective trial in eight different centers, showed a statistically significant decline in surgical mortality and a reduction in associated surgical complications from a rate of 11% to 7% after introduction of the checklist. All health care settings should establish surgical safety checklist operated by trained personnel within a culture of safety [14].

## Conclusion

As identified in the “To Err Is Human” report, adverse events and errors are not the result of “a few bad apples” or inattentive individuals. Adverse events and errors are symptoms of a process in healthcare which is not running smoothly. This is especially true in Sub-Saharan Africa, where the errors that propagated an initial error into eventual harm may not be detected, or are detected but not corrected along the way [14]. An increased attention to disclosure of adverse events and errors will give a significant boost to efforts to improve health care delivery in the surgical patient. As surgeons in Sub-Saharan Africa have done in the past, and continue to do so today, we must search for and embrace all measures that will insure the health and well-being of the patients we serve [6].
